# Are PDRNs the Next Magic Ingredient? From Cosmetic Hype to Regenerative Bioengineering

**DOI:** 10.1111/jocd.71263

**Published:** 2026-10-27

**Authors:** Raíssa Nunes dos Santos

**Affiliations:** ^1^ Graduate Program in Biosciences Federal University of Health Sciences of Porto Alegre (UFCSPA) Porto Alegre Brazil

## 1 Opinion

Regenerative medicine has repeatedly been transformed by new classes of biomolecules, from hyaluronic acid and platelet‐rich plasma to exosomes. More recently, polydeoxyribonucleotides (PDRNs) have attracted increasing interest in regenerative dermatology, with market analyses projecting substantial growth in the PDRN and skin‐booster markets [1]. Yet beyond their growing commercial presence lies a fundamental scientific question: what defines a biologically active PDRN?

PDRNs have been associated with regenerative effects involving adenosine A2A receptor signaling and downstream processes related to angiogenesis, extracellular matrix remodeling, collagen synthesis, and tissue repair [2]. However, evidence from cell and animal models should be distinguished from demonstrated clinical efficacy in humans. Preclinical findings provide mechanistic and proof‐of‐concept evidence but do not establish cosmetic effectiveness, optimal dosing, durability, or long‐term safety.

This uncertainty becomes particularly relevant when considering the biological heterogeneity of PDRNs. Commercial PDRNs are still differentiated primarily by their biological origin rather than their molecular characteristics, while the extent to which biological origin contributes to regenerative activity remains incompletely understood. Indeed, comparative studies of fragmented DNA from human placenta, porcine placenta, and salmon sperm have reported source‐associated differences in biological responses, with DNA methylation proposed as one potential contributor [3]. Thus, the assumption that PDRNs from different sources are functionally equivalent remains unproven. Rather than representing a single therapeutic entity, PDRNs may constitute a heterogeneous family of biomolecules whose activity depends on structure–function relationships.

This knowledge gap is also evident in the current innovation landscape. Most commercial PDRNs are derived from salmonid reproductive tissues, reflecting historical availability, established production processes, and mature supply chains. To characterize this technological landscape, we conducted an exploratory analysis of Google Patents, identifying 388 unique patent families among 404 retrieved records. Patent activity increased markedly after 2021, with 76.5% of the identified families prioritized between 2021 and 2025 (Figure S1). The landscape was also geographically concentrated, with China and South Korea accounting for 83.8% of the identified patent families, while Brazil was not represented among the recorded jurisdictions (Figure S2). At the technological level, formulation/composition and production‐process claims predominated, suggesting that innovation remains largely focused on extraction, processing, and formulation rather than on diversification of biological sources or molecular engineering (Figure S3). The leading assignees were exclusively based in China or South Korea, with no Brazilian institution or company among those with six or more patent families (Table S1). Screening of source‐ and technology‐related keywords further revealed limited diversification beyond salmonid‐derived PDRNs, including a small number of plant‐derived approaches but no patents involving Brazilian or other tropical South American species (Table S2). The complete search strategy and dataset are provided in Supplementary Information and Data S1. Taken together, these findings suggest that PDRN innovation remains largely tied to established biological sources and conventional extraction and formulation approaches, while comparatively little attention has been given to the molecular features that may govern biological activity. This gap invites a conceptual shift: from asking where PDRNs come from to asking which molecular features make them biologically active. Such a structure–function perspective could redefine PDRNs not as source‐defined products, but as a broader class of regenerative polynucleotides (Figure 1).

**FIGURE 1:**
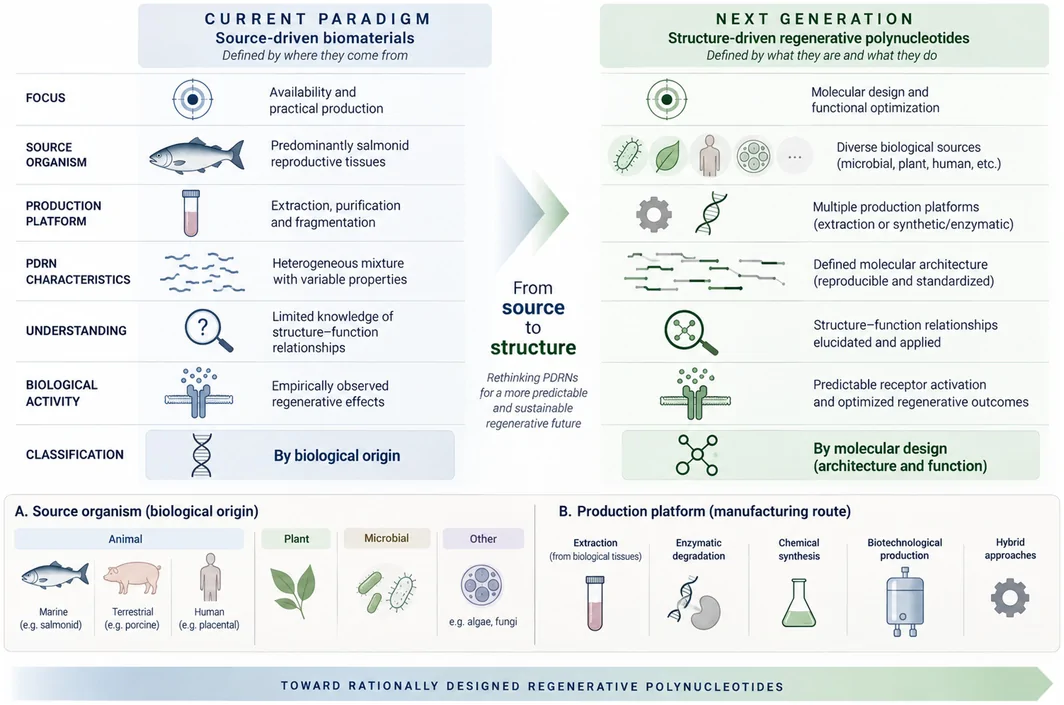
From source‐driven biomaterials to structure‐driven regenerative polynucleotides. Conceptual schematic illustrating a proposed transition from PDRNs defined primarily by biological origin toward regenerative polynucleotides characterized by molecular architecture, structure–function relationships, and predictable biological activity. The proposed paradigm shifts the focus from empirically extracted DNA toward rationally designed biomaterials with defined molecular properties and biological functions.

Importantly, emerging evidence challenges a strictly source‐dependent view of PDRN activity. Microbial‐derived PDRNs have demonstrated regenerative effects in preclinical models, while DNA fragments derived from human placental mesenchymal stem cells have also shown regenerative activity involving A2A receptor signaling [4, 5]. Together, these findings suggest that regenerative activity may extend across biologically diverse sources, while also underscoring the complexity of the relationship between molecular characteristics, concentration, and biological response. Rather than indicating that source is irrelevant, these observations suggest that biological origin may interact with molecular features and formulation parameters to shape PDRN activity.

## 2 Conclusions: Are PDRNs the Next Magic Ingredient?

Probably not and that may be precisely what makes them compelling. Rather than following transient marketing trends, the next generation of regenerative dermatology may depend on understanding the molecular determinants of PDRN bioactivity and translating them into polynucleotides with predictable functions. The question may therefore no longer be whether PDRNs are the next “magic ingredient,” but whether regenerative medicine is moving toward a future in which regenerative polynucleotides are designed rather than merely extracted. In that future, their value may be defined less by where they come from than by what their molecular architecture enables them to do.

## Funding

The author has nothing to report.

## Conflicts of Interest

The author declares no conflicts of interest.

## Supporting information


**Figure S1:** Number of PDRN‐related patent families by priority year (*n* = 388; 13 records with unavailable priority date excluded). Patent activity increased markedly after 2021, with 297 of 388 families (76.5%) priority‐dated between 2021 and 2025.
**Figure S2:** Distribution of PDRN‐related patent families by jurisdiction of publication (*n* = 388). China and South Korea jointly account for 325 families (83.8% of the total); Brazil does not appear among the recorded jurisdictions.
**Figure S3:** Distribution of PDRN‐related patent families by technology category, based on title‐level keyword matching (*n* = 388, nonexclusive categories). Formulation/composition and production‐process claims dominate the landscape, consistent with an extraction‐ and formulation‐centered innovation pattern rather than a mechanism‐ or source‐diversification‐centered one.
**Table S1:** Assignees with six or more patent families in the dataset. All leading assignees are China‐ or South Korea‐based; no Brazilian institution or company appears among assignees at any frequency.
**Table S2:** Occurrence of source‐ and technology‐related keywords in patent titles (*n* = 388). *Nine families mention “plant” in the title; six of these are genuine plant‐derived PDRN/PDRN‐precursor patents (production from plant tissue or plant cell culture), filed 2021–2024 by two South Korean assignees using non‐Brazilian species (e.g., rose, Gynostemma pentaphyllum). None involve Brazilian or other tropical/native South American species.

**Data S1:** Data contains all 388 patent families analyzed in this study (Google Patents export, retrieved April 10, 2026), including patent identifiers, titles, assignees, inventors, priority/filing/publication/grant dates, and result links.

## Data Availability

The data that support the findings of this study are available on request from the corresponding author. The data are not publicly available due to privacy or ethical restrictions.
