# Supplementary material for: Are PDRNs the Next Magic Ingredient? From Cosmetic Hype to Regenerative Bioengineering
Source: J Cosmet Dermatol. 2026 Oct 27;25(11):e71263. doi: 10.1111/jocd.71263 (PMC13714336; doi:10.1111/jocd.71263)
Supplement: Supplementary file 1 [file JOCD-25-e71263-s001.docx]

**Supporting Information 1**

**Patent landscape analysis of polydeoxyribonucleotide (PDRN) technologies**

Raíssa Nunes dos Santos

*Supplementary to: “Are PDRNs the Next Magic Ingredient? From Cosmetic Hype to Regenerative Bioengineering”*

# **S1. Patent search strategy**

Patent documents were retrieved from Google Patents on April 10, 2026, using the keyword “PDRN” combined with the classification query CL=(PDRN).

Database: Google Patents (patents.google.com).

Search date: April 10, 2026.

Inclusion criteria: all patent documents returned by the query above, with no restriction on jurisdiction, patent status, or filing type (application or granted patent).

Deduplication: records were deduplicated by patent family identifier; documents corresponding to the same family across offices were merged.

Initial search: 404 records → deduplication → 388 unique patent families → analysis by jurisdiction, assignee, priority year, and title-level technology/biological-source keywords.

The complete dataset is available as Supplementary Dataset S1.

## **Growth over time**

*Figure S1. Number of PDRN-related patent families by priority year (n = 388; 13 records with unavailable priority date excluded). Patent activity increased markedly after 2021, with 297 of 388 families (76.5%) priority-dated between 2021 and 2025.*

## **Geographic distribution**

*Figure S2. Distribution of PDRN-related patent families by jurisdiction of publication (n = 388). China and South Korea jointly account for 325 families (83.8% of the total); Brazil does not appear among the recorded jurisdictions.*

Patent titles were screened for non-exclusive technology categories to characterize the dominant application areas within the dataset (a single title may match more than one category).

*Figure S3. Distribution of PDRN-related patent families by technology category, based on title-level keyword matching (n = 388, non-exclusive categories). Formulation/composition and production-process claims dominate the landscape, consistent with an extraction- and formulation-centered innovation pattern rather than a mechanism- or source-diversification-centered one.*

## **Leading assignees**

| **Top assignees (≥6 families)** | **Families** |
| --- | --- |
| Runhui Biotech (润辉生物技术) | 8 |
| Ruijiming Biotech (瑞吉明生物科技) | 8 |
| Saimeng Biotech group (赛萌系) | 7 |
| Unisolve Co., Ltd. (유니솔브) | 7 |
| Biyan Medical Technology (碧研医疗) | 6 |
| Fengjin Beauty Technology (丰金美业) | 6 |

*Table S1. Assignees with six or more patent families in the dataset. All leading assignees are China- or South Korea-based; no Brazilian institution or company appears among assignees at any frequency.*

Emerging technological opportunities

| **Keyword (patent title)** | **Occurrences** |
| --- | --- |
| Salmon | 6 |
| Recombinant | 6 |
| Marine | 3 |
| Trout | 1 |
| Placenta | 1 |
| Stem cell | 1 |
| Algae | 1 |
| Fermentation | 1 |
| Plant / plant-derived | 9* |
| Yeast | 0 |
| Probiotic | 0 |
| Fibroblast | 0 |
| Aquaculture | 0 |
| Tilapia | 0 |
| Pirarucu | 0 |
| Tambaqui | 0 |
| Brazilian native species (any) | 0 |

*Table S2. Occurrence of source- and technology-related keywords in patent titles (n = 388). *Nine families mention “plant” in the title; six of these are genuine plant-derived PDRN/PDRN-precursor patents (production from plant tissue or plant cell culture), filed 2021–2024 by two South Korean assignees using non-Brazilian species (e.g., rose, Gynostemma pentaphyllum). None involve Brazilian or other tropical/native South American species.*

These results indicate that technological innovation remains strongly concentrated on salmon-derived extraction, formulation, and production-process claims, while several alternative source and production strategies — aquaculture by-products, yeast, probiotics, and fibroblast-based platforms are absent from the dataset entirely. Plant-derived PDRN is not itself an unclaimed category: it is already the subject of at least six South Korean patent families using non-native, non-Brazilian plant sources. The unclaimed space identified by this analysis is therefore narrower and more specific than “plant-derived PDRN” in general: no patent family in the dataset involves a Brazilian or broader tropical South American species, and no assignee in the dataset is based in Brazil.

# **Supplementary discussion**

The present analysis demonstrates that innovation in the PDRN field is concentrated on optimizing extraction, purification, and formulation from salmonid tissues rather than on investigating molecular engineering strategies or diversifying biological sources. Growth in filing activity since 2021 has occurred almost entirely within this same technological and geographic base, rather than alongside a broadening of source diversity.

Recent literature describing microbially produced and non-salmonid animal-derived PDRN (main text, refs. 5–6) suggests that the technological landscape may soon diversify beyond salmon. The patent record analyzed here shows that plant-derived PDRN has already entered this diversification, with production-process patents filed in South Korea since 2021 using ornamental and traditional-medicine plant species.

# **Supplementary Dataset S1**

Supplementary Dataset S1 contains all 388 patent families analyzed in this study (Google Patents export, retrieved April 10, 2026), including patent identifiers, titles, assignees, inventors, priority/filing/publication/grant dates, and result links.
